# Amyotrophic Lateral Sclerosis, a Multisystem Pathology: Insights into the Role of TNF*α*

**DOI:** 10.1155/2017/2985051

**Published:** 2017-09-10

**Authors:** Massimo Tortarolo, Daniele Lo Coco, Pietro Veglianese, Antonio Vallarola, Maria Teresa Giordana, Gabriella Marcon, Ettore Beghi, Marco Poloni, Michael J. Strong, Anand M. Iyer, Eleonora Aronica, Caterina Bendotti

**Affiliations:** ^1^Department of Neuroscience, IRCCS Istituto di Ricerche Farmacologiche Mario Negri, Milan, Italy; ^2^ALS Research Center, Dipartimento di Biomedicina Sperimentale e Neuroscienze Cliniche (BioNeC), University of Palermo, Palermo, Italy; ^3^Department of Neuroscience, University of Turin, Turin, Italy; ^4^Department of Medical, Surgical and Health Sciences, University of Trieste, Trieste, Italy; ^5^DAME, University of Udine, Udine, Italy; ^6^Cell Biology Research Group, Robarts Research Institute, London, ON, Canada; ^7^Department of Neuropathology, Academisch Medisch Centrum, Amsterdam, Netherlands

## Abstract

Amyotrophic lateral sclerosis (ALS) is considered a multifactorial, multisystem disease in which inflammation and the immune system play important roles in development and progression. The pleiotropic cytokine TNF*α* is one of the major players governing the inflammation in the central nervous system and peripheral districts such as the neuromuscular and immune system. Changes in TNF*α* levels are reported in blood, cerebrospinal fluid, and nerve tissues of ALS patients and animal models. However, whether they play a detrimental or protective role on the disease progression is still not clear. Our group and others have recently reported opposite involvements of TNFR1 and TNFR2 in motor neuron death. TNFR2 mediates TNF*α* toxic effects on these neurons presumably through the activation of MAP kinase-related pathways. On the other hand, TNFR2 regulates the function and proliferation of regulatory T cells (Treg) whose expression is inversely correlated with the disease progression rate in ALS patients. In addition, TNF*α* is considered a procachectic factor with a direct catabolic effect on skeletal muscles, causing wasting. We review and discuss the role of TNF*α* in ALS in the light of its multisystem nature.

## 1. Introduction

Amyotrophic lateral sclerosis (ALS) is a progressive and fatal adult motor neuron disease (MND), known since 1864, but still mysterious as concerns the mechanism of onset and the ineluctable progression characterized by increasing muscular atrophy, with loss of strength, paralysis, and death [1–3]. Death is due to respiratory failure and occurs typically 3–5 years after diagnosis, although in some cases, survival is longer than two decades [4]. Usually, the disease starts focally with subtle weakness of the limb or bulbar muscles and then spreads, progressing to paralysis of almost all skeletal muscles. The pathology encompasses distant biological systems including the brain, spinal cord, and muscle/neuromuscular junctions. It is now becoming clear that ALS also involves other nonneuromuscular systems which may have substantial roles in driving the degenerative process and/or influencing the rate of disease progression, hence the prognosis. These systems include the peripheral immune system, innate and adaptive, and are influenced by the overall metabolic status [5, 6].

Nearly 90% of all ALS cases arise spontaneously, while the remaining 10% are linked to genetic mutations, mostly inherited as a dominant trait. Along with the well-known mutations in the Cu^2+^/Zn^2+^ superoxide dismutase (SOD1) gene [7], discovered more than two decades ago as a unique gene mutation specifically linked to ALS, twelve other ALS genes have been discovered in the last ten years. These genes can be grouped into several categories based on their protein function and their involvement in (i) protein homeostasis, such as optineurin [8], valosin-containing protein [9], ubiquilin 2 [10], and TBK1 [11]; (ii) RNA metabolism and function such as TAR DNA-binding protein 43 (TDP-43), fused in sarcoma/translocated in liposarcoma (FUS/TLS) [12], C9orf72 [13, 14], matrin 3 [15], and angiogenin [16]); (iii) cytoskeletal dynamics of motor axons such as dynactin subunit 1 [17], profilin 1 [18], and tubulin alpha-4 A chain [19]; (iv) mitochondrial function such as CHCHD10 [20]; and (v) regulation of inflammation such as TBK1 [11]. This genetic variability explains the complexity of the disease in which heterogeneous mechanisms converge towards a common pathogenesis. These mechanisms include alterations in RNA processing and stability, dysfunction in proteostasis and protein quality control, mitochondrial dysfunction and increased oxidative stress, defects of the cytoskeletal dynamics in the motor axons and distal terminals, synaptic impairment, and neuroinflammation [21].

Neuroinflammation is a typical hallmark of ALS, detectable in the nervous system and peripheral biological fluids. While the disease progression in ALS is a result of slow and progressive dysfunction and loss of motor neurons, other nonneuronal cells in the central nervous system (CNS) and peripheral nervous system (PNS), including the immune cells, play crucial roles [22, 23]. Microglia and astroglia proliferation and activation are prominent histological features in the spinal cord and motor cortex of ALS patients and have been detected *in vivo* by positron emission tomography during the course of the disease [24, 25]. Infiltrates of macrophages and T lymphocytes have been reported in both the CNS and PNS of ALS patients and in animal models, testifying the direct involvement of the immune system [6, 26–28].

TNF*α* is one of the major proinflammatory cytokines, with a central role in the initiation and orchestration of immunity and inflammation. TNF*α* participates in local and systemic inflammation with pleiotropic actions including both pro- and anti-inflammatory functions. It acts through two main receptors, the p55 TNF*α* receptor (TNFR1) and the p75 TNF*α* receptor (TNFR2), that differ in their binding affinity for TNF*α*, expression pattern, and downstream signal transduction cascades [29]. This cytokine has been implicated in motor neuron death occurring in ALS patients and animal models [30–33]. However, the controversial results regarding its role in governing the progression of the disease in ALS mice [34, 35] and the lack of efficacy of the treatment with anti-TNF*α* therapeutics in patients [36] have reduced the general interest about its possible relevance in the pathology. Nevertheless, a recent gene expression study using next-generation RNA sequencing (RNAseq, Illumina) analysis in postmortem cervical spinal cord from sporadic ALS patients identified significant elevation of inflammatory processes with TNF*α* as a major regulatory molecule [37]. TNF*α* was also detected as one of the main candidate hubs in a gene coexpression network in the fibroblasts of ALS patients with C9orf72 mutation, underlying its potential contribution to the altered immune response in ALS [38]. There is also fresh evidence that the two receptors TNFR1 and TNFR2 may act in opposite directions in motor neuron degeneration, suggesting new perspectives in identifying specific potential therapeutic targets [39, 40].

These recent discoveries, together with the increasing evidence of the role of the immune system as a primary event in ALS pathology, prompted us to re-examine the evidence linking TNF*α* with the etiology of the disease. This review discusses the potential role of TNF*α* at the intersection of various cellular and molecular mechanisms associated with the pathological alterations in ALS, not only in the nervous system but also in relation to the muscle wasting and metabolic changes that characterize the disease.

## 2. TNF*α*, TNF*α* Receptors, and Related Intracellular Pathways in ALS

TNF*α* is a 26 kDa cytokine expressed by activated monocytes/macrophages, microglia, and activated natural killer and T cells and also by nonimmune cells like astrocytes, endothelial cells, fibroblasts, and neurons [41]. Once synthesized as a 233-amino acid-long type II transmembrane monomeric protein, it is transferred into the membrane, forming a stable homotrimer. Membrane-bound TNF*α* (mTNF*α*) is then cleaved by TNF*α*-converting enzyme (TACE), also called ADAM17, which releases a soluble form of TNF*α* (sTNF*α*). This circulates throughout the body displaying its potent endocrine function and its ability to act at distant physiological sites.

Both soluble and membrane TNF*α* are biologically active, and a number of factors, including cell status and stimuli, control the balance between the two forms. Their signal transduction involves binding with two transmembrane receptors (TNFRs), TNFR1 and TNFR2, which are differently expressed and regulated and control different signaling pathways. While TNFR1 is constitutively expressed in almost all cells, TNFR2 is mainly expressed in lymphocytes and other immune cells and is induced by different cell stimulations [41]. TNFR1 binds to both sTNF*α* and mTNF*α* while TNFR2 preferentially interacts with mTNF*α* and is believed to play an important role in localized signaling during cell-to-cell interactions, possibly also through “reverse signaling” processes [41, 42]. The cytoplasmic tail of TNFR1 contains a death domain that is missing in TNFR2.

Although initially TNFR1 activation was considered to be primarily involved in the cytotoxic and apoptotic effects of TNF*α*, while TNFR2 stimulation was related exclusively to cell survival and proliferation, now, it is becoming clear that TNFR2 can also induce cell death, directly or indirectly. For instance, through a cell-to-cell interaction, the binding between mTNF*α* and TNFR2 can induce tumor and neuronal cell death [39, 43, 44].

The complex and divergent roles of TNF*α*-induced TNFR signaling in apoptosis and inflammation have been described elsewhere [45]. Of particular interest in the present review is the role of TNFRs in modulation of the signaling mediated by the mitogen-activated protein kinase (MAPK) cascade, implicated in neuroinflammatory and neurodegenerative mechanisms [45]. Moreover, membrane-integrated TNF*α* can receive stimulation from TNFR binding, resulting in a “reverse signaling”, that activates signals into the mTNF*α*-bearing cell. This process enables a two-way communication in cell-to-cell contact, possibly contributing to the plasticity of the ligand-receptor systems and facilitating the fine-tuning of the immune response [41, 46]. As discussed later, this mechanism can also play a significant role in the interaction between motor neurons and neighboring glial cells in ALS models [39].

**Figure 1:**
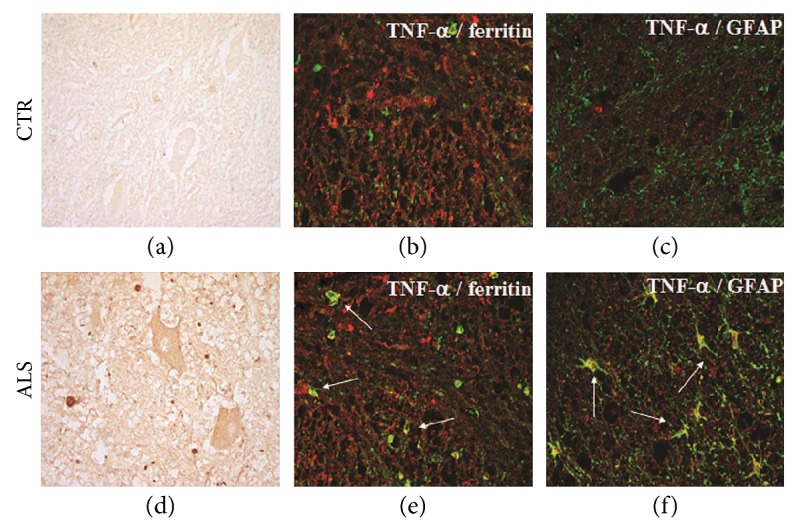
Immunolocalization of TNF*α* in the spinal cord of ALS patients. Expression in the cytosol of motor neurons was weak in the control spinal cord (a). ALS patients showed increased TNF*α* labeling in the motor neurons (d). Laser scanning confocal micrographs of immunofluorescence for TNF*α* (red) and ferritin (microglia, green) (b, e) or GFAP (astrocytes, green) (c, f) show upregulation of TNF*α* in the glial cells of ALS patients (e, f, arrows) compared to those of controls (b, c). Magnification: 40x. Spinal cords from sporadic ALS (five males and three females) and control patients with nondegenerative or nonneurological diseases (three males and three females) were used. Controls were patients with one of the following: cardiac failure, chronic sepsis, and CNS or non-CNS tumor. The mean age at death was 60 years (range 52–69) for ALS patients and 64 years (range 55–75) for controls. Duration of illness in ALS cases ranged between 10 and 60 months. All cases were autopsied within 8 to 15 hours from death. Tissues were fixed in formalin and embedded in paraffin.

The actual contribution of TNF*α* to the pathophysiology of ALS remains highly controversial on account of the pleiotropic nature of this cytokine and related pathways. Numerous studies have demonstrated altered homeostasis of the TNF*α* system in ALS patients and in mouse models of the disease. Elevated levels of TNF*α* were reported in the blood [30, 31, 47] and cerebrospinal fluid (CSF) [48] of ALS patients. While the high plasma levels, which positively correlate with the illness duration [49], may derive from a large proportion of TNF*α*-positive cells in the epidermis and in some blood vessels and glands of patients, the high CSF levels may come from the upregulation of TNF*α* in motor neurons and reactive microglial/astroglial cells seen in the spinal cord of individuals affected by sporadic ALS (Figure 1).

**Figure 2:**
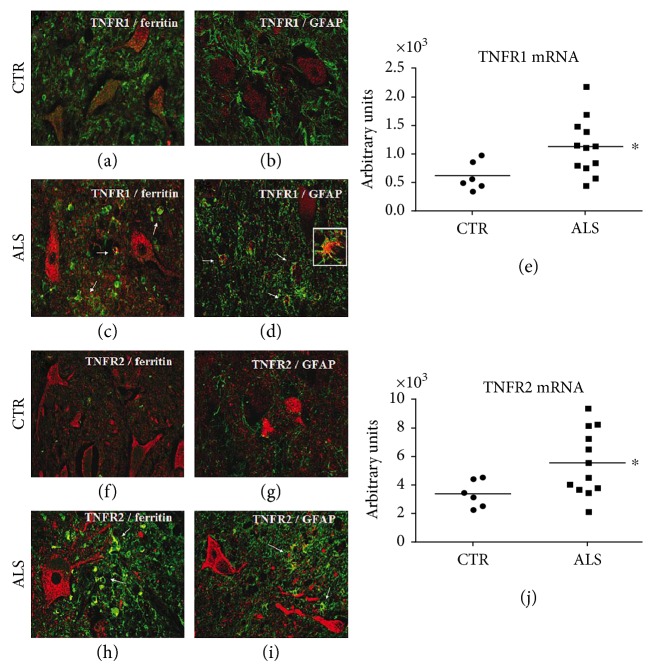
Expression of TNFR1 and TNFR2 in the spinal cord of ALS patients. Laser scanning confocal micrographs of immunofluorescence for TNFR1 (red) and ferritin (microglia, green) or GFAP (astrocytes, green) showed higher expression of TNFR1 in the glial cells of ALS patients (c, d, arrows) than those of controls (a, b). Magnification: 40x and 60x (inset, d). Immunofluorescence for TNFR2 (red) and ferritin (microglia, green) or GFAP (astrocytes, green) showed higher levels of TNFR2 in the glial cells of ALS patients (h, i, arrows) than those of controls (f, g). Magnification: 40x. (e, j) Quantitative RT-PCR analysis of TNFR1 and TNFR2 mRNA levels in the lumbar spinal cord of ALS patients (*n* = 12) and controls (*n* = 6). Data were analyzed by *t*-test (^∗^*p* < 0.05 ALS versus controls).

High levels of TNF*α* were also detected in the spinal cord of SOD1^G93A^ transgenic mice before symptom onset [33, 40, 50–52] and in motor neurons and microglia of symptomatic wobbler mice, another model of motor neuron disorder [53, 54], suggesting a prominent role of this cytokine in the development of the disease. Thalidomide and lenalidomide, two immune-modulatory drugs that induce the degradation of TNF*α* mRNA injected presymptomatically, lowered TNF*α* expression in the spinal cord, reduced motor neuron loss, improved the motor deficit, and increased the survival of SOD1^G93A^ mice [34]. However, the constitutive deletion of the gene coding for TNF*α* in the same mouse model did not change the lifespan or reduce motor neuron degeneration [35]. This approach, which completely abolished TNF*α* expression during development, probably activated compensatory mechanisms through which the mutant SOD1 could exert its toxicity. Not only TNF*α* but also both its receptors TNFR1 and TNFR2 were markedly upregulated in the spinal cord of presymptomatic SOD1^G93A^ [40, 51, 52] and symptomatic wobbler [53] mice. This was also evident in sporadic ALS patients. In fact, we found significant upregulation of TNFR1 and TNFR2 transcripts in the homogenate of the lumbar spinal cord from ALS patients compared to nonneurological controls, and this correlated with an increased immunoreactivity of both receptors in the reactive astrocytes and dystrophic microglial cells (Figure 2). Thus, it appears that while TNF*α* is overproduced and probably secreted by microglia, astrocytes, and neurons under stress conditions [55] (Figure 1), its receptors are upregulated only in the glial cells.

Recently, studying astrocyte-spinal neuron cocultures from SOD1^G93A^ mice, we noted that upregulation of mTNF*α* specifically in motor neurons was detrimental as treatment with thalidomide or anti-TNF*α* antibody completely rescued motor neurons in this experimental paradigm. Interestingly, the reduction in TNFR2, but not in TNFR1, in either astrocytes or neurons as well as treatment with anti-TNFR2 antibody completely abolished the motor neuron death [39]. Since in an astrocyte-free spinal neuronal culture, the soluble TNFR2 did not rescue the motor neurons, but rather killed them, we suggested that the detrimental effect of mTNF*α* on motor neurons was exerted through a cell contact interaction between the TNFR2 exposed on astrocytes and mTNF*α* on motor neuron membranes, through a reverse signaling mechanism. Thus, we hypothesize that SOD1^G93A^ can render motor neurons sensitive to astroglial TNFR2, by increasing the expression of mTNF*α* in the cell membrane. The detrimental role of TNFR2 was confirmed *in vivo* in SOD1^G93A^ mice knockout for the receptor, where there was a partial but significant protection of motor neurons in the lumbar spinal cord and spared neuromuscular junctions (NMJ) and conserved morphology of tibialis muscle fibers [39]. However, these positive effects did not result in any improvement of motor performance or survival. Motor neurons and their axons, even if partially preserved, were probably compromised in their function since sciatic nerves expressed lower levels of acetylated *α*-tubulin and had increased accumulation of phospho-TDP-43, two indices of axonal dysfunction that might explain the lack of clinical improvement. In contrast, the ablation of TNFR1 significantly increased the loss of motor neurons and accelerated the disease progression in SOD1^G93A^ mice [40], suggesting a protective role of TNF*α* signaling via TNFR1. The authors demonstrated that this protective effect was mediated by TNFR1 expressed by astrocytes, stimulated by endogenous TNF*α* to release the glial-derived neurotrophic factor (GDNF), a potent neurotrophic factor for motor neurons. These studies clearly indicate that TNFR1 and TNFR2 expressed by astrocytes can act in opposite directions in governing the survival of motor neurons under stressful conditions linked to mutant SOD1. Therefore, with a view to possible therapeutic development, new strategies are needed to activate TNFR1 and inhibit TNFR2 specifically in astrocytes.

Not so clear, instead, is the role of the upregulation of both TNFRs in motor neurons of SOD1^G93A^ mice during the development of the disease [52]. Recently, we demonstrated by a transcriptomic analysis of laser-dissected motor neurons from SOD1^G93A^ mice that the synthesis of both TNFR1 and TNFR2 is significantly increased at the onset and in the symptomatic stage of the disease, supporting the idea that intraneuronal cross-talk between TNFR1 and TNFR2 could play a role in motor neuron death [56]. It was reported that only the combined deletion of both TNFR1 and TNFR2 receptors prevented motor neuron death after facial nerve axotomy in adult mice [57]. However, since TNFR1 and TNFR2 were deleted in neurons and in nonneuronal cells, it is hard to define the specific role of each receptor and cell type in the protective response.

TNFR1 is generally believed to mediate TNF*α*-induced cell death signaling, but there is increasing evidence of the neuroprotective activity of this receptor in neurons, as reviewed by Probert [58]. For example, Taoufik et al. showed that neuroprotection induced by erythropoietin and Vascular endothelial growth factor (VEGF) against oxygen-glucose deprivation and NMDA excitotoxicity depended significantly on the presence of TNFR1 in cortical neurons [59]. In addition, microglia-derived TNF*α* exerted neuroprotective effects through TNFR1 on cerebral ischemia [60]. In contrast, TNFR2, which is commonly considered to promote survival signaling through direct recruitment of TRAF2 and the activation of PI3K and NF-*κ*B pathways [41], had toxic effects on motor neurons in our *in vitro* and *in vivo* models of ALS [39]. Sipe and colleagues also reported a detrimental role of TNFR2 on neuronal cells [44]. They showed that the induction of TNFR2 expression on the surface of N1E neuroblastoma cells caused cell death through the binding of mTNF*α* exposed by adjacent cells, through a cell-to-cell contact mechanism.

Microglia are considered key players in the neuroinflammation that accompanies the development of ALS. Recent evidence indicates that TNF*α* released by activated microglia is essential in inducing the neurotoxic A1 phenotype in astrocytes that lose their trophic function [61] and A1 astroglial cells were found in autoptic tissues of patients with different neurodegenerative diseases, including ALS [61]. Importantly, extracellular SOD1^G93A^ has been reported to activate, through the CD14-TLR2 pathway, a neurotoxic phenotype in microglia characterized by the increased release of proinflammatory cytokines, including TNF*α* [62]. Further, activation of the ionotropic purinergic receptor P2X7 in SOD1^G93A^ microglia leads to the production of significantly high levels of TNF*α*, which exert a neurotoxic effect on motor neuron cultures [63].

LPS activation of NF-*κ*B in microglial cells expressing mutant TDP-43 is associated with the production of proinflammatory cytokines such as TNF*α*, IL-6, and IFN*γ* [64]. The release of TNF*α* by microglia can also generate a self-sustained autocrine loop via TNFR1 stimulation, inducing a neuroinflammatory activated phenotype [65], which leads to a state of chronic neuroinflammation detrimental to motor neurons. Thus, if TNFR1 in astrocytes is needed for a protective effect on ALS-injured motor neurons, its presence in the microglia may be toxic by exacerbating neuroinflammation. The specific ablation of TNFR2 in microglia induced a proinflammatory and more invasive phenotype of these cells, reducing their homeostatic functions and accelerating the onset of the disease in an EAE model of multiple sclerosis [66].

Oligodendrocytes are another type of a nonneuronal cell susceptible to TNF*α*'s action, and their implication in the pathogenic mechanisms of ALS has been only recently reported [67]. Oligodendrocytes are severely affected during the disease, and they degenerate before motor neuron death. In the attempt to compensate for oligodendrocyte loss, progenitor cells become highly proliferative but fail to reach the fully differentiated state [68]. As a result, motor fibers in the spinal cords of mouse models and ALS patients show evident signs of demyelination [27, 67]. TNF*α* interferes with oligodendrocyte differentiation and causes their death [69], effects mediated by TNFR1 [70, 71]. In contrast, the activation of TNFR2 which is mainly expressed in oligodendrocyte progenitor cells protects these cells from oxidative stress [71]. Therefore, also in oligodendrocytes, a diverging action of the two TNF*α* receptors emerges, as in microglia and astrocytes. However, their relative contributions to the action of these cells during the development of ALS require further investigation.

This evidence suggests that the opposite effects of TNFRs on survival or death of motor neurons depend on the cell population involved. For example, while the inhibition of TNFR1 in microglia and oligodendrocytes can be protective for motor neurons, the response becomes detrimental if the TNFR1 is inhibited in astrocytes. The opposite is expected with TNFR2 inhibition.

These data illustrate the difficulty of effectively regulating the action of TNF*α*/TNFR signaling on motor neuronal degeneration. Therefore, information on the downstream pathways is required to identify specific targets and conditions that allow the balance between beneficial and detrimental effects of TNF*α*, to finally obtain neuroprotection.

**Figure 3:**
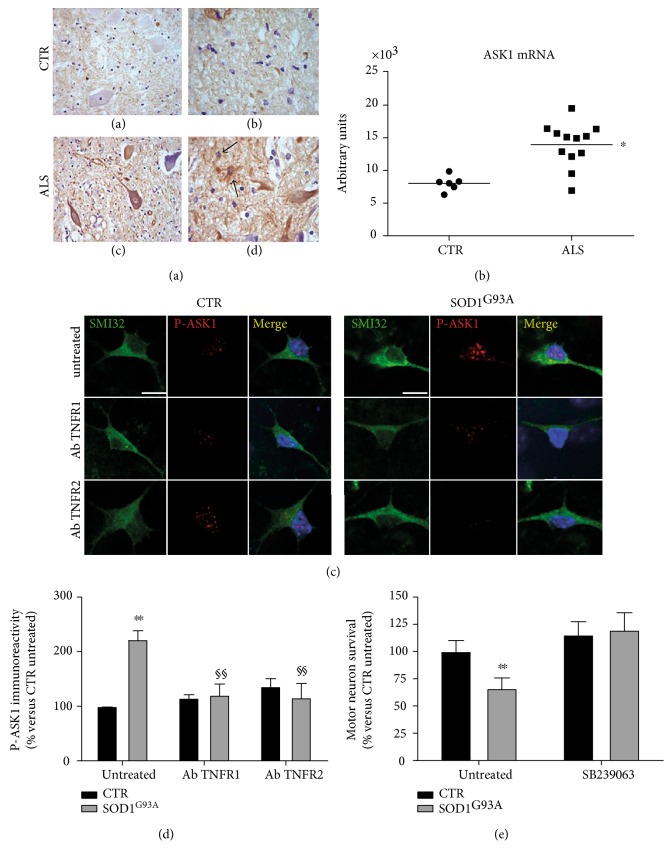
Phosphorylated ASK-1 (P-ASK1) in the spinal cord of ALS patients. The control spinal cord shows a weak cytoplasmic staining for P-ASK1 in the anterior horn motor neurons (a). ALS patient spinal cords had increased immunoreactivity for P-ASK1 in the cell bodies and proximal neurites of anterior horn motor neurons (c). P-ASK1 immunostaining was more expressed in the glial cells (astrocytes and microglia) of ALS patients (d, arrows) than those of controls (b). Magnification: 40x. (e) Quantitative RT-PCR analysis of ASK1 mRNA levels in the lumbar spinal cord of ALS patients (*n* = 12) and controls (*n* = 6). Data were analyzed by *t*-test (^∗^*p* < 0.05 ALS versus controls). (f) Immunocytochemistry for phosphorylated ASK-1 (P-ASK1, red) in astrocyte-spinal neuron cocultures expressing SOD1^G93A^ and nontransgenic control (CTR). Large motor neurons expressing SOD1^G93A^ identified by SMI32 labeling (green) and morphological criteria (maximum diameter and shape) showed greater immunoreactivity for P-ASK1 than those of controls. Three-day treatment with neutralizing antibodies anti-TNFR1 (AbTNFR1) or anti-TNFR2 (AbTNFR2) reduced immunoreactivity for P-ASK1 in the transgenic motor neurons. Magnification: 60x; scale bar: 10 *μ*m. The graph (g) shows the quantification of P-ASK1 immunoreactivity. The bar graph represents mean ± SD (% of untreated control) of three independent experiments. Data were analyzed by two-way ANOVA followed by Tukey's multiple comparison test (^∗∗^*p* < 0.01 compared to untreated CTR; ^§§^*p* < 0.01 compared to SOD1^G93A^). (h) Effect of the P-p38MAPK inhibitor SB239063 on motor neuron viability in astrocyte-spinal cord cocultures expressing SOD1^G93A^. SB239063 totally protected transgenic motor neurons in SOD1^G93A^ cocultures after six days *in vitro*. Data were analyzed by two-way ANOVA followed by Tukey's multiple comparison test (^∗∗^*p* < 0.01 compared to untreated CTR). All experiments with cell cultures were done as previously described [45].

The difficulty in interpreting the TNF*α* system's effects also lies in the variety of intracellular pathways it can activate, as widely reviewed [41, 72, 73]. We previously reported that the high levels of TNFRs in the spinal cord of mutant SOD1 mice were associated with activation of signaling involving MKK3-6, MKK4, ASK1, and phosphorylated p38MAPK (P-pMAPK) in motor neurons and glial cells [52, 74]. Phospho-p38MAPK accumulation was also found in the spheroid-like inclusions observed in human ALS motor neurons [75]. We have recently confirmed that ASK1 mRNA and immunoreactivity are also elevated in the spinal cord of ALS patients (Figure 3). This supports the hypothesis based on the SOD1^G93A^ mouse study of the involvement of the TNFRs/ASK1/p38MAPK axis in spinal motor neuron degeneration [52].

To investigate further the interaction between TNF*α*, its receptors, and the ASK1/p38MAPK pathway in ALS, we examined the effect of selective TNFR1 or TNFR2 antagonist-neutralizing antibodies in primary astrocyte-spinal neuron cocultures expressing SOD1^G93A^. Unexpectedly, the significant increase in phospho-ASK1 (P-ASK1) immunoreactivity in transgenic large motor neurons was prevented by inhibition of each of TNFRs separately (Figure 3). These results suggest that the ASK1/p38MAPK pathway may, at least in this cellular paradigm, be a convergent target for both the TNF*α* receptors, but since only the inhibition of TNFR2 preserved motor neuron from death [39], the significance of the response to TNFR1 inhibition remains to be clarified. There is ample evidence that the ASK1/p38MAPK pathway activation is detrimental to ALS motor neurons. For example, the deletion of ASK1 reduced motor neuron loss and prolonged the lifespan of mutant SOD1 mice [76, 77]. In addition, the inhibition of p38MAPK with the selective antagonist SB239063 completely prevented motor neuron death in SOD1^G93A^ cocultures (Figure 3). This agrees with a recent report that CIIA mitigated SOD1^G93A^-induced cytotoxicity by inhibiting the activation of ASK1/p38MAPK signaling [78]. In addition, previous studies showed significant protection of motor neurons and proximal axons in SOD1^G93A^ mice after administration of the p38MAPK inhibitor semapimod [79], although this effect was not accompanied by an increase in survival.

These data suggest the importance of the TNFR2/ASK1/p38MAPK pathway in the mechanisms of motor neuron degeneration and point out the growing evidence of a dissociation between the protection of motor neurons and the lack of an effect on mouse survival. This is probably because various cellular components are involved in ALS pathology, like muscles and the immune system, that can react differently to therapeutic agents or genetic manipulation.

## 3. TNF*α* and the Immune System in ALS

Evidence is accumulating that the immune system, both innate and adaptive, is critical in the pathology of ALS [6, 23, 26, 28]. In the CNS microglia and macrophages, the local components of the innate immune response become activated in the affected tissues in response to priming events, for example, misfolded and aggregated proteins, mitochondrial dysfunction, or impaired axonal transport, triggering neuroinflammation. This may lead to a state of chronic activation of the innate and adaptive immune system as indicated by the infiltration of macrophages, dendritic cells, and T lymphocytes (CD4^+^ and CD8^+^) in the central [6, 80] and peripheral nervous system [81] of SOD1^G93A^ mice at different stages of the disease, as well as in postmortem tissues from patients [23, 26].

Although initially neuroinflammation was considered detrimental in ALS, this idea was challenged by the demonstration of a dual role of immunity with protective and toxic phases during the disease progression: an early M2/Th2 protective phase characterized by stable disease and a late M1/Th1 phase characterized by rapid disease progression [82, 83]. During the early stable phase, the immune system is protective with glia and T cells, especially M2 microglia/macrophages and T helper 2 cells (Th2)/regulatory T cells (Treg), providing anti-inflammatory factors that sustain motor neuron viability. This phase is characterized by enhanced IL-10 and IL-4 expression in the spinal cord and accumulation of FoxP3, a marker of suppressive Treg cells in the spinal cord, lymph nodes, and blood of mutant SOD1 mice, followed by an overall decrease as the disease progresses [84]. Interestingly, ALS patients have low levels of circulating Tregs [24], with an inverse correlation between the rate of disease progression and the percentages of circulating Treg and FoxP3 [85]. Therefore, low Treg and FoxP3 mRNA levels were proposed as accurate biomarkers predicting rapid disease progression [86]. In addition, passive transfer of Tregs from donor ALS mice during the early slow phase or by ex vivo cell expansion extended the early phase and prolonged survival of recipient SOD1^G93A^ mice [87, 88], demonstrating the neuroprotective properties of these cells. The enhanced neuroprotection by Treg cells was attributed to the release of anti-inflammatory cytokines with subsequent maintenance of M2 microglia and suppression of effector T cells (Teffs). Thus, increasing the Tregs in patients at the early stages of the pathological process might be a promising therapeutic approach to hamper the disease progression [86].

Among the factors that exert strong activity on Tregs, TNF*α* plays a major role, although with divergent effects. In fact, TNF*α* can either promote Treg proliferation and expansion or limit the suppressive capacity [89]. Therefore, depending on which effect prevails, TNF*α* may exert either protective (anti-inflammatory) or pathogenic (proinflammatory) activity. TNFR2, but not TNFR1, is expressed by human and mouse CD4^+^/FoxP3 Treg, in the steady and activated state [90], and it has been suggested that the coexpression of CD25 with TNFR2 could mark the cells with the most suppressive capacity [91].

In addition to the ability to induce proliferation of Tregs, TNF*α* may also control their suppressive function. TNF*α* strongly downregulated FoxP3 expression in human Treg cells [92] and inhibited its phosphorylation [93]. Therefore, TNF*α* and its receptors, particularly TNFR2, might potentially be able to tip the balance between pathogenic Teff cells and protective Treg cells in both directions, depending on synergy with other inflammatory factors. For example, IL-6 which is overexpressed in ALS at the later phases also inhibits the generation of FoxP3^+^ Treg cells induced by TGF-beta [94] and may act in synergy with TNF*α* to reduce Treg function. Therefore, considering the high expression of FoxP3 in mutant SOD1 mice at the early stage of the disease [88], it is reasonable to assume that an initial TNF*α*-TNFR2 interaction may be critical for the early neuroprotective activity, while in the late phase, the increased levels of both TNF*α* and IL6 may synergize to suppress the protective Treg. However, when we examined the levels of FoxP3 in the spinal cord of SOD1^G93A^ mice lacking the TNFR2 receptors, we did not find difference from SOD1^G93A^/TNFR2^+/+^ mice. Therefore, Treg modulation may not be a primary mechanism in the motor neuron protection derived from TNFR2 inhibition.

On the other hand, we confirmed that Treg levels in the spinal cords of SOD1^G93A^ mice at an advanced symptomatic stage were significantly lower than those in the spinal cords of nontransgenic littermates, in line with the hypothesis of a reduction in these cells during the late M1 phase of disease progression. TNFR2 deficiency in mice has been reported to lower the overall cytokine production and proliferation of CD8^+^ T cells [95]. Therefore, we cannot exclude that the general depletion of TNFR2 could have reduced the later detrimental proinflammatory effect of the M1/T1 phase in both the central and peripheral compartments, preventing the massive loss of motor neurons and neuromuscular junction in SOD1^G93A^ mice at the symptomatic stage. It remains to be explained why the protection of the neuromuscular system in SOD1^G93A^/TNFR2-knockout mice was not associated with an improvement in the disease course. Conceivably, some overall proinflammatory activity of TNF*α*, through TNFR1, or other proinflammatory cytokines like INF*γ* and IL-6 may have a negative impact on the muscle wasting and general metabolic changes seen in these mice during symptom progression, masking the benefit resulting from the protection of the motor neurons and NMJs.

## 4. TNF*α* and Muscle Wasting in ALS

TNF*α* is a cell signaling protein responsible for several metabolic derangements leading to muscle wasting [96]. It promotes a wasteful metabolic process in muscles, leading to increased protein degradation and consequent loss of skeletal muscle mass. This occurs through a pathway in which reactive oxygen species (ROS) and NF-*κ*B are the early mediators of a cascade leading to protein degradation through activation of the ubiquitin proteasome pathway (UPP). The intracellular ROS induced by the activity of TNF*α* leads to activation of IKKb, making NF-*κ*B available for translocation to the nucleus where it activates the expression of genes that regulate the UPP. In doing this, TNF*α* not only promotes the loss of proteins but also becomes responsible for transcription regulation, cell cycle progression, and antigen presentation in the muscles [97]. This process mediated by TNFR1, not TNFR2, is involved in muscle protein degradation and wasting [98].

The progressive wasting of skeletal muscle is the main cause of the loss of strength, motor disability, paralysis, and death in ALS. Although the dismantlement of neuromuscular junctions is considered the primary cause of muscle atrophy, ALS patients and mouse models all show clear muscle dysmetabolism/hypermetabolism in relation to disease progression [99]. Proteomic analysis of gastrocnemius muscle from SOD1^G93A^ mice with clear signs of motor impairment reported an accumulation of ROS together with an increase in NF-*κ*B and impaired mitochondrial respiratory function. Unlike in the spinal cord, no accumulation of mutant SOD1 was found in the skeletal muscle of SOD1^G93A^ mice during the disease progression [100]. This suggests that the induction of NF-*κ*B in the muscle of mutant ALS mice may activate the machinery for misfolded protein degradation, that is, UPP and autophagy, in clear contrast with what happens in the spinal cord [101]. It is still not known whether the NF-*κ*B activation in ALS mouse muscle is mediated by TNF*α*. However, an active inflammatory response, with elevated levels of TNF*α*, was recently reported in the hindlimb muscles of SOD1^G93A^ rats [102], suggesting that TNF*α*-mediated inflammation might contribute to the muscle wasting in SOD1 mice and perhaps in ALS patients too.

TNF*α* also plays an important role in the metabolic alterations like altered lipid and carbohydrate metabolism and insulin resistance that are typical of cachexia syndrome [96]. Cachexia is a clinical condition much akin to ALS. The syndrome is characterized by weight loss due to loss of both muscle and adipose tissues but with disproportionate muscular wasting in patients with cancer or other chronic systemic disorders like inflammatory diseases (sepsis, rheumatoid arthritis, and HIV) or cardiac, renal, and respiratory insufficiency [103].

Patients with ALS are generally lean and lose body mass, muscle mass, and fat as the disease progresses [99]. An association of diabetes and insulin resistance with ALS has been suggested in several studies, although there are controversial opinions on whether this association is protective or detrimental to the disease development and progression [5]. Insulin resistance has been considered an important mechanism for the muscle wasting in cachexia as it increases proteolysis by increasing the expression of UPP components [104], and TNF*α* is responsible for this [96]. Therefore, considering the high TNF*α* levels found in biological fluids of ALS patients [30, 31, 48], we cannot exclude a potential role of this cytokine in the metabolic derangements associated with the disease.

## 5. TNF*α* and Glutamate-Mediated Excitotoxicity

Another mechanism through which TNF*α* can induce or otherwise affect the motor neuron degeneration in ALS involves its action on excitotoxicity. Excitotoxicity is the result of excessive activation of glutamate receptors which may be due to failure in neurotransmitter clearance from the synaptic cleft or increased postsynaptic sensitivity to glutamate. The first evidence of an involvement of excitotoxicity in ALS was the markedly high glutamate levels in the cerebrospinal fluid of ALS patients [105]. This was later attributed to altered glutamate clearance from the synaptic cleft due to the reduced expression and function of the Na^+^-dependent glutamate transporter EAAT2 (GLT-1 in rodents) in ALS patients [106] and mouse models [107, 108]. This transporter, which is responsible for removing up to 94% of glutamate from the synaptic cleft, is present on the astrocytic processes that envelop the synapse [109].

Overactivation of amino-3-hydroxyl-5-methyl-4-isoxazole-propionate (AMPA) receptors is considered the main cause of excitotoxicity for motor neurons. Spinal motor neurons possess several Ca^2+^-permeable AMPA receptors under basal conditions, and this high density may increase susceptibility to degeneration [110–112]. AMPA receptor calcium permeability is largely determined by the GluR2 subunit which is posttranscriptionally edited, changing a codon encoding glutamine to one encoding arginine (Gln/Arg) in the second transmembrane domain, making the receptor complex impermeable to calcium. Reduced GluR2 subunits have been reported in spinal motor neurons of ALS patients [110] and SOD1^G93A^ mutant mice [113], making them highly permeable to calcium and more susceptible to excitotoxic insults. In keeping with this, the overexpression or deficit of GluR2 subunits in SOD1^G93A^ mice improved or accelerated the disease progression, respectively [111, 114], while different AMPA receptor antagonists slowed the disease progression and prolonged the survival of SOD1 mutant mice [113, 115]. Unfortunately, in patients, the noncompetitive AMPA antagonist talampanel failed to improve the disease course [116].

Thus, multifaceted evidence suggests that increased glutamatergic neurotransmission may contribute to the motor neuron neurodegeneration in ALS even if inhibitors of the excitotoxicity, except for riluzole, have failed in clinical trials [117]. TNF*α* is the first endogenous mediator known to influence the excitotoxicity and the extracellular levels of glutamate by both enhancing its release and reducing its reuptake [118]. The first evidence comes from studies in human neuronal cultures showing that subtoxic doses of TNF*α* and AMPA became neurotoxic when combined [119]. Hermann and colleagues then demonstrated that nanoinjections of nontoxic doses of TNF*α* and kainate combined in rat spinal cord led to neuronal cell death which was reversed by an AMPA receptor antagonist [120].

TNF*α* is also a critical component of the regulatory system controlling synaptic plasticity [121], synaptic strength, and excitability by modifying AMPA receptor trafficking. This occurs through a deficiency of GluR2 with a consequent increase in Ca^2+^ permeability [122]. In line with this, TNF*α* can potentiate AMPA receptor-mediated excitotoxicity on lumbar spinal motor neurons by inducing rapid membrane reassortment with a heavy presence of Ca^2+^-permeable AMPA receptors [123]. TNF*α* was also reported to downregulate astrocytic EAAT2/GLT-1 expression, and this effect was mediated through the NF-*κ*B binding to the EAAT2 promoter [124, 125]. Another mechanism through which TNF*α* may promote excitotoxicity has been demonstrated in cultured astrocytes through the binding of TNF*α* to TNFR1, which raises intracellular calcium, followed by glutamate exocytosis [126]. In mouse primary microglia too, TNF*α* can induce excitotoxicity in an autocrine manner through the TNFR1 pathway, by promoting microglial release of glutamate [127].

Finally, besides increasing the surface expression of glutamate receptors, TNF*α* also induces endocytosis of GABA-A receptors, reducing their inhibitory action [128]. Thus, the exacerbation of excitotoxicity mediated by TNF*α* can occur at different levels: (i) by increasing the expression of calcium-permeable AMPA receptors in motor neurons, (ii) by reducing glutamate reuptake by astrocytes, and (iii) by increasing glutamate release from microglia through the TNFR1 pathway. On the basis of these data, it would be reasonable to propose the inhibition of TNF*α*/TNFR1 signaling as a neuroprotective therapeutic approach. However, as discussed earlier, TNFR1 activation in astrocytes is vital for the release of GDNF and the protection of motor neurons. Therefore, the overall manipulation of TNFR1 receptors in the CNS may have opposite effects on motor neuron survival, making this approach impracticable for pharmacological therapy in ALS. Only the targeted therapeutic approaches using gene therapy or engineered molecules to selectively interfere with the expression and activity of this receptor in a cell-specific manner might effectively improve the disease progression.

## 6. Anti-TNF*α* Therapy in ALS

Although there is no doubt that TNF*α* plays a role in ALS, as reviewed here and elsewhere, different anti-inflammatory treatments intended to lower its levels have achieved only partial improvement, no effect, or even exacerbation of the symptoms in animal models and patients. For example, minocycline, which reduces the synthesis of TNF*α* and other inflammatory mediators, delayed the disease progression in mutant ALS mice [129] but had harmful effects on patients in a phase III randomized clinical trial [130]. Similar results were obtained with thalidomide, a more specific TNF*α* synthesis inhibitor, which partially relieved the motor deficit and prolonged the survival of SOD1^G93A^ mice [34] but caused adverse effects and did not effectively affect the disease progression of ALS patients in a phase II clinical trial [36].

There are several possible reasons for the different responses in animal models and patients. A major issue is that in the mouse models treatment is often given before the symptom onset; on the contrary, in patients, the disease is usually already advanced by the time of diagnosis and treatment, so the motor neurons are already markedly compromised. In fact, when minocycline was given to SOD1 mutant mice after the disease onset, it did not affect survival and exacerbated the inflammatory milieu around motor neurons [131].

It is likely—as evidenced from this review—that TNF*α*/TNFR signaling has many different roles at the different stages of disease and only targeting it in the initial phase may prevent the massive loss of motor neurons and rapid progression of the pathology. Therefore, it becomes essential to identify early diagnostic markers in patients to allow prompt pharmacological intervention with anti-TNF*α* therapies. Although thalidomide and lenalidomide completely rescued the spinal motor neuron degeneration in SOD1 mutant mice, the gains in motor performance and in lifespan were only transient and modest, suggesting that other parts of the neuromuscular system were not preserved by anti-TNF*α* therapy. This is probably explained by the pleiotropic action of TNF*α* and the different contributions of its receptors in a multicellular disease involving motor neurons, astrocytes, microglia, infiltrated immune cells, and muscles, as discussed earlier. What is needed now is novel, highly specific, and selective anti-TNF*α* therapeutic approaches to ensure the efficacy and safety of treatments in ALS.

A wide range of different biologic molecules and immunomodulatory drugs have been developed to inhibit the action of TNF*α* and/or interfere with their receptor activity as a therapeutic strategy in inflammatory peripheral disease and autoimmune disorders [41, 45]. They include FDA-approved biologics that bind to both mTNF*α* and sTNF*α*, preventing the binding and activation of TNF*α* receptors. However, the pleiotropic nature of TNF*α* and the nonspecific activity of these molecules lead to serious side effects such as lymphoma, congestive heart failure, demyelinating disease, and infections [132].

Despite these drawbacks and the poor penetration of the blood-brain barrier by these agents, clinical trials with etanercept, a genetically engineered Fc fusion protein generated from the extracellular domain of human TNFR2 which blocks both sTNF and mTNF, have been conducted in patients with Alzheimer disease (AD), with significant cognitive and behavioral improvements [133, 134]. On the contrary, treatment of multiple sclerosis (MS) patients with lenercept, a dimeric TNFR1 extracellular domain fused to a human IgG1, was stopped during a phase II randomized study, due to an increase in the frequency, duration, and severity of MS attack [135]. The failure was probably caused by the nonspecific inhibition of both sTNF*α* and mTNF*α* which acting through TNFR1 and TNFR2, respectively, can mediate nerve demyelination or remyelination through different pathways, as reviewed by McCoy and Tansey [41].

To date, no clinical trials have been conducted in ALS using these anti-TNF*α* biologics. However, case reports have been published showing patients developing ALS after long treatments with anti-TNF*α* molecules. Dziadzio and collaborators described a patient with rheumatoid arthritis who was diagnosed with ALS after receiving infliximab for five years [136]. Subsequently, several other ALS cases treated with TNF*α* blockers (adalimumab, etanercept, infliximab, and others) for rheumatoid arthritis and ankylosing spondylitis were reported [137–139]. Although a direct causal relationship between ALS development and TNF*α* blockers has not been established [140], it cannot be excluded that interfering with the TNF*α* system for a long time can worsen or even trigger motor neuron degeneration, given the potential neuroprotective effect of TNF*α* that we have described in this review. All these evidences strongly suggest that general inhibition of TNF*α* cannot achieve any notable benefit because it can interfere with different mechanisms with opposite activity at the same time.

The recent demonstration of the protective function shown by astroglial TNFR1 through the production of GDNF [40] in an ALS mouse model and the neurotoxic activity of TNFR2 carried by astrocytes and neurons [39] mediated by the ASK1/p38MAPK pathway suggests that a future targeted therapeutic strategy should selectively potentiate the TNFR1 response and antagonize TNFR2 or its downstream signaling, in a cell-specific manner.

## 7. Summary and Conclusions

**Figure 4:**
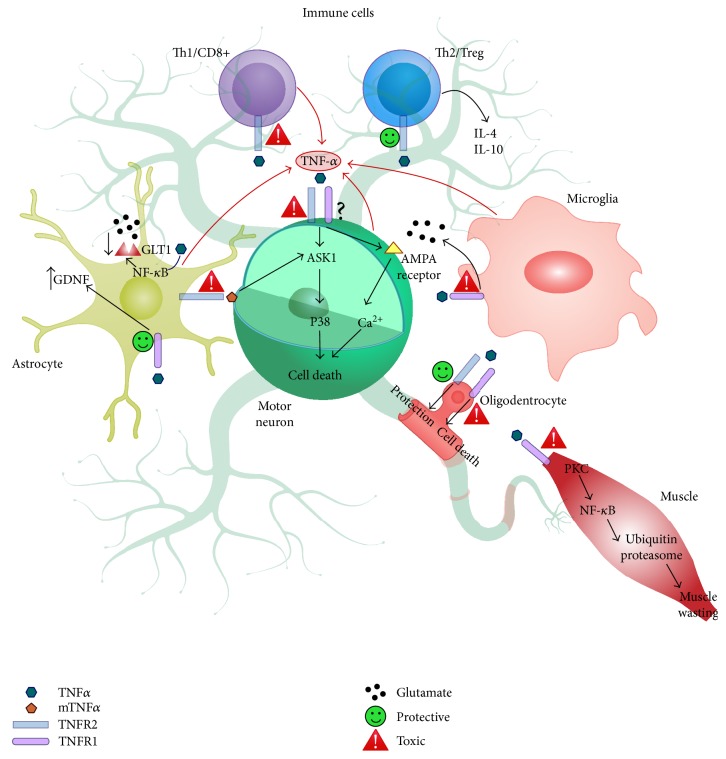
Potential mechanisms by which TNF*α* and its receptors TNFR1 and TNFR2 contribute to the neuropathology in ALS. Both neuroprotective and neurotoxic functions are proposed for TNFR1 and TNFR2, depending on the cell type involved.

Neuroinflammation and the immune response, both innate and adaptive, are considered to actively contribute to the initiation and propagation of ALS, a pathology that involves different cell types in the neuromuscular, glial, and peripheral immune systems. TNF*α* is the major cytokine instrumental in governing these mechanisms (Figure 4). However, its action can influence the pathophysiology either positively or negatively depending on the type of receptor and cell involved throughout all stages of the disease (Table 1). For example, while through its action on TNFR2, TNF*α* may activate Treg cells that regulate the initial neuroprotective M2/T2 phase of the disease by releasing anti-inflammatory cytokines; the active interaction between this receptor localized on the surface of astrocytes and the mTNF*α* on motor neurons may trigger a cascade leading to motor neuron death. Thus, TNFR2 inhibition should be driven specifically to the astrocytes to prevent motor neuron degeneration. At the same time, strategies aimed at increasing the expression and/or activity of TNFR2 in the Tregs and olygodendrocytes may promote an immunomodulatory function, prolonging the M2 phase and promoting remyelination. The role of TNFR1 in regulating the disease progression appears far more complicated. While the activation of TNFR1 on astrocytes induces GDNF release protecting motor neurons, its activation also provokes a reduction in the glutamate transporter GLT1 causing an increase in extracellular glutamate with potential excitotoxic effects. Excitotoxicity may also result from the activation of TNFR1 on microglia due to excessive glutamate release from these cells. Finally, increased TNFR1 signaling activity in the CNS could lead to the degeneration of oligodendrocytes with consequent axonal demyelination, while its activation in muscle may lead to excessive protein degradation, hence skeletal muscle wasting and atrophy.

These findings seem to indicate an overall detrimental effect of TNFR1 on ALS pathology. However, the fact that mutant SOD1 mice with a constitutive depletion of TNFR1 have a shorter lifespan suggests that the activity of this receptor in the various cell types may be balanced during ALS course, possibly depending on the levels of TNF*α* and/or on the activity of the antithetic TNFR2 receptors in the same cells.

Given the rapid development of new curative strategies based on cell-specific targeting, for example, through gene therapy or engineered molecules, we can envisage the possibility of more selective anti-TNF*α* agents that may regulate TNFR expression and downstream signaling in specific cell populations. The role of the TNF*α*/TNFR pathway in ALS calls for further investigations.

## Figures and Tables

**Table 1:** Effects of the activation of TNFRs on different cell types.

Cell types	Activation of TNFR1	Activation of TNFR2
Motor neurons		Cell death [39]
Astrocytes	Release of GDNF [40], increase in intracellular Ca^2+^, and release of glutamate [123]	Death of cocultured motor neurons [39]
Microglia	Activation of proinflammatory phenotype [63] and release of glutamate [124]	Induction of anti-inflammatory phenotype [64]
Oligodendrocytes	Cell death and reduced differentiation [68, 69]	Protection from oxidative stress [71]
CD8^+^ T cells		Increased cytokine production and proliferation [93]
Treg cells		Proliferation and suppressing function control [89]
Muscle cells	Protein degradation and muscle wasting [96]	

## Abbreviations

ALS: Amyotrophic lateral sclerosis
AMPA: Amino-3-hydroxyl-5-methyl-4-isoxazole-propionate
CNS: Central nervous system
CSF: Cerebrospinal fluid
FDA: Food and Drug Administration
GFAP: Glial fibrillary acidic protein
LPS: Lipopolysaccharides
MAPK: Mitogen-activated protein kinase
mTNF*α*: Membrane TNF*α*
NF-*κ*B: Nuclear factor kappa-light-chain-enhancer of activated B cells
NMDA: N-Methyl-D-aspartate
NMJ: Neuromuscular junctions
PNS: Peripheral nervous system
SOD1: Superoxide dismutase 1
ROS: Reactive oxygen species
sTNF*α*: Soluble TNF*α*
TDP-43: TAR DNA-binding protein 43
Teff: T effector cells
TNF*α*: Tumor necrosis factor *α*
TNFR1: Tumor necrosis factor receptor 1
TNFR2: Tumor necrosis factor receptor 2
Treg: Regulatory T cells
VEGF: Vascular endothelial growth factor.
